# Comparison of Different Semi-Automated Bioreactors for In Vitro Propagation of Taro (*Colocasia esculenta* L. Schott)

**DOI:** 10.3390/plants10051010

**Published:** 2021-05-19

**Authors:** Eucario Mancilla-Álvarez, Juan Antonio Pérez-Sato, Rosalía Núñez-Pastrana, José L. Spinoso-Castillo, Jericó J. Bello-Bello

**Affiliations:** 1 Facultad de Ciencias Biológicas y Agropecuarias, Universidad Veracruzana, Veracruz C.P. 94945, Mexico; euca_man90@hotmail.com (E.M.-Á.); ronunez@uv.mx (R.N.-P.); 2 Colegio de Postgraduados Campus Córdoba, Km. 348 de la Carretera Federal Córdoba-Veracruz, Veracruz C.P. 94500, Mexico; pantonio@colpos.mx (J.A.P.-S.); jlspinoso@gmail.com (J.L.S.-C.); 3 CONACYT-Colegio de Postgraduados Campus Córdoba, Km. 348 de la Carretera Federal Córdoba-Veracruz, Veracruz C.P. 94500, Mexico

**Keywords:** acclimatization, chlorophyll, in vitro multiplication, semi-automated bioreactors, stomatal content

## Abstract

Taro is important for its nutritional content, medicinal use, and bioethanol production. The aim of the present study was to compare different semi-automated bioreactors (SABs) during in vitro multiplication of *C. esculenta*. The SABs used were temporary immersion bioreactors (TIBs), SETIS™ bioreactors and ebb-and-flow bioreactors; semi-solid culture medium was used as a control treatment. At 30 d of culture, different developmental variables, determination of chlorophyll, stomatal content, and survival percentage during acclimatization were evaluated. SABs increased the shoot multiplication rate relative to the semi-solid medium; however, the SETIS™ bioreactor showed the highest shoot production, with 36 shoots per explant, and the highest chlorophyll content. The stomatal index was higher in the semi-solid medium compared to the SABs, while the percentage of closed stomata was higher in the SABs than in the semi-solid culture medium. The survival rate during acclimatization showed no differences among the culture systems assessed, obtaining survival rates higher than 99%. In conclusion, the SETIS™ bioreactor showed the highest multiplication rate; however, other bioreactor alternatives are available for semi-automation and cost reduction for micropropagation of *C. esculenta*.

## 1. Introduction

Taro (*Colocasia esculenta* L. Schott) is a tropical plant cultivated because its corms have nutritional benefits and industrial use (bioethanol production). Moreover, tarin a glycoprotein obtained from taro, is used for medicinal purposes, such as antitumoral, anti-hyperglycemic and anti-hyperlipidemic activities [1,2]. This species is propagated both sexually and asexually; sexual propagation is rare and produces seeds with low genetic quality [3], while traditional asexual propagation by corms is seasonal and can cause the spread of pests and diseases [4]. In vitro propagation of *C. esculenta* through semi-automated bioreactors (SABs) is an alternative for the production of high-quality genetic and phytosanitary clones [5]. SABs are designed for in vitro multiplication of plants by tissue, cell or organ in liquid culture medium with a programmed temporary immersion (TI) [6]. The advantage of SABs is that they increase multiplication rates, allow greater availability of culture medium components and reduce production costs [7,8,9]. Micropropagation in SABs promotes photosynthesis and respiration, both caused by an increase in chlorophyll synthesis and stomatal functionality [10,11].

Different SABs designs exist for commercial plant micropropagation. The bioreactor design has effect on in vitro plant stress physiology, growth and differentiation [11,12,13]. The most commonly used bioreactors in micropropagation include the recipient for automated temporary immersion (RITA^®^, Vitropic, St. Mathieu de Tréviers, FR), temporary immersion bioreactors (TIBs) [14], the ebb-and-flow bioreactor [15], the monobloc advance temporary immersion system (MATIS^®^ bioreactor, Cid-plastiques, Valergue, FR), the PlantForm^TM^ bioreactor [16] and the SETIS™ bioreactor [17]. It is important to assess the efficiency of different SABs for each species due to considerations regarding their multiplication rate, availability, and cost. The efficiency of different SABs during micropropagation has been evaluated in anthurium (*Anthurium andreanum*) [12], helleborine orchid (*Epipactis flava* Seidenf) [18], sugarcane (*Saccharum* spp.) [13] and apple (*Malus domestica*) [19]. Taro micropropagation has been carried out in semi-solid medium [20] and in a TIB [5]; however, other bioreactors need to be validated as an alternative for micropropagation. The aim of the present study was to compare the efficiency of the TIB, SETIS™, ebb-and-flow bioreactor, and semi-solid medium culture systems for in vitro multiplication of *C. esculenta*.

## 2. Results

### 2.1. Effect of Culture Systems on In Vitro Multiplication

When evaluating the effect of different culture systems on in vitro multiplication, significant differences were observed among the variables (shoots per explant, shoot length (cm), leaves per shoot, roots per explant, root length (cm) and survival (%)) evaluated during the in vitro multiplication (Table 1).

**Figure 1:**
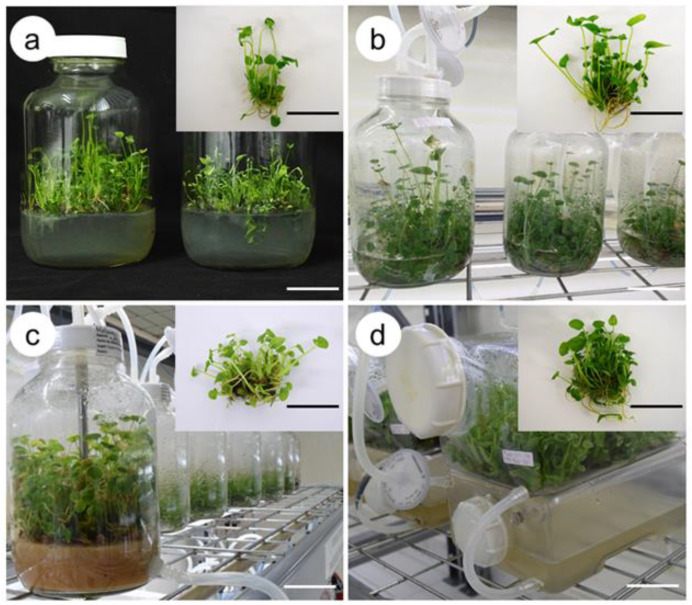
Comparison of culture systems of taro (*Colocasia esculenta*) shoot multiplication after 30 days of culture. (**a**) Semi-solid medium, (**b**) temporary immersion bioreactor, (**c**) ebb-and-flow bioreactor (**d**) SETIS™ bioreactor. Black and white bar = 5 cm.

The highest multiplication rate was observed in the SETIS^TM^, with 36 shoots, followed by the TIB and ebb-and-flow bioreactor, with 21.70 and 20 shoots per explant, while the lowest number of shoots was obtained in the semi-solid medium with 6.10 shoots per explant (Figure 1). For shoot length, the longest shoot length was obtained in the TIB and SETIS^TM^ culture systems, with 5.50 and 4.50 cm, respectively, whereas the shortest shoot length occurred in the ebb-and-flow bioreactor, with 2.50 cm. Regarding the number of leaves per shoot, the TIB and SETIS^TM^ culture systems showed the highest number of leaves with 3.70 and 3.60 leaves, respectively, whereas the lowest number of leaves was observed in the semi-solid medium with 2.70 leaves per shoot and the ebb-and-flow system with 3.10 leaves. The highest number of roots was observed in the SETIS^TM^ culture system, with 41.60 roots per explant, whereas the lowest number of roots was observed in the semi-solid medium and TIB, with 5.80 and 2.50 roots, respectively. For the variable root length, the shoots generated in the semi-solid medium had the longest root length, with 5.60 cm, and the shortest root length was obtained in the ebb-and-flow bioreactor system, with 1.40 cm.

### 2.2. Chlorophyll Content in Different Culture Systems

**Figure 2:**
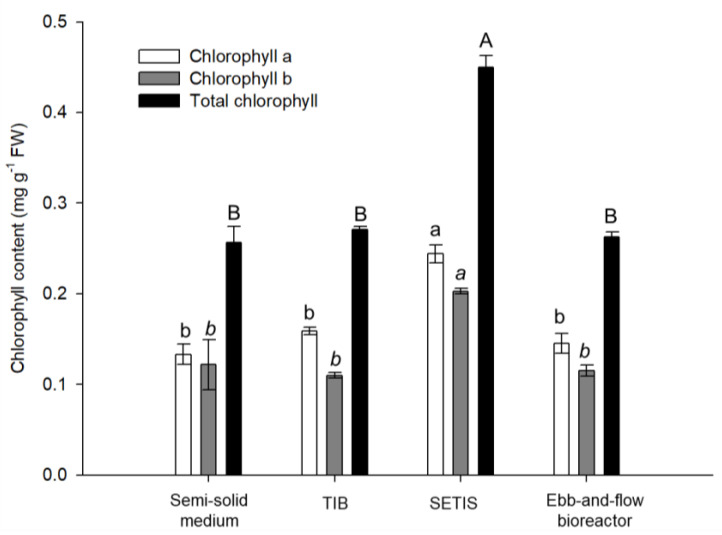
Chlorophyll content in different culture systems of in vitro shoots of *Colocasia esculenta* at 30 days of culture. Means ± standard error within a bar followed by the same letter are not significantly different (Tukey, *p* ≤ 0.05). TIB: temporary immersion bioreactor; SETIS: SETIS™ bioreactor.

The chlorophyll (chl) contents showed significant differences among the culture systems. The highest chl a, b, and total chl contents were observed in the SETIS™ bioreactor, with 0.44 mg g^−1^ FW, whereas the lowest chl contents were observed in the TIB, ebb-and-flow bioreactor, and semi-solid medium culture systems, obtaining values between 0.25 and 0.26 mg g^−1^ FW (Figure 2).

### 2.3. Stomatal Content

**Figure 3:**
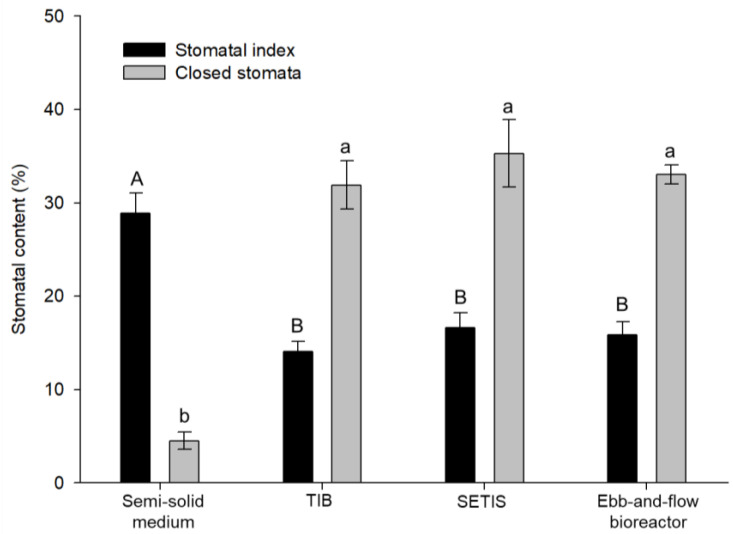
Stomatal content in different culture systems of in vitro shoots of *Colocasia esculenta* at 30 days of culture. Means ± standard error within a bar followed by the same letter are not significantly different (Tukey, *p* ≤ 0.05). TIB: temporary immersion bioreactor; SETIS: SETIS™ bioreactor.

**Figure 4:**
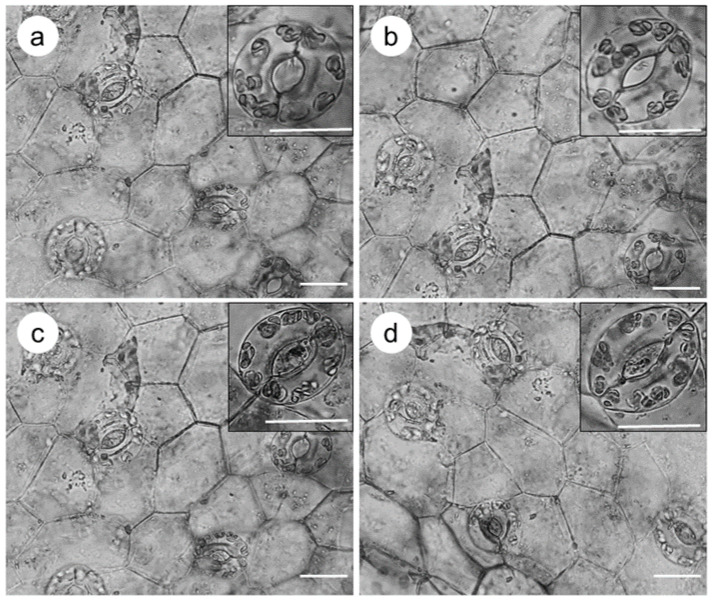
Stomatal index (SI) and open stomata (OE) of taro (*Colocasia esculenta*) in different culture systems at 30 days of culture. (**a**) Semi-solid medium, (**b**) TIB: temporary immersion bioreactor, (**c**) ebb-and-flow bioreactor, (**d**) SETIS™ bioreactor. White bar = 100 µm.

The stomatal index (SI) and closed stomata (% CS) showed significant differences among the culture systems (Figure 3). Similar stomatal morphology was observed in all evaluated systems; however, the highest SI was in semi-solid systems, with 28.85%, whereas the temporary immersion systems exhibited a lower SI, ranging from 14.05% to 16.63%. For the % CS, shoots cultured in temporary immersion had the highest % CS, between 31% and 35%. Shoots cultured in a semi-solid system had the lowest % CS, with 4.51% (Figure 4).

### 2.4. Acclimatization Ex Vitro

**Figure 5:**
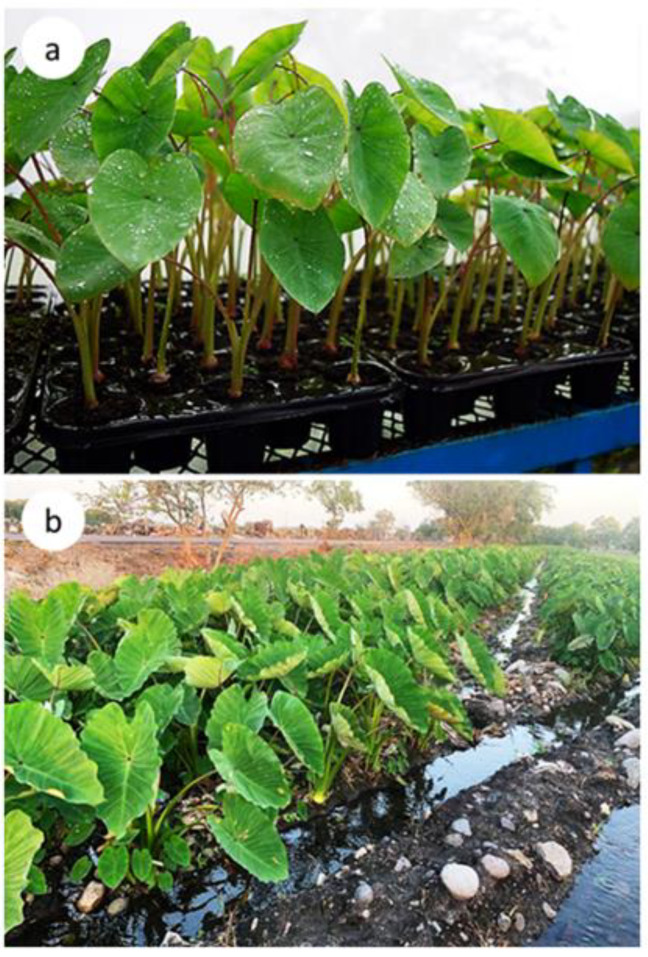
Ex vitro seedlings of *Colocasia esculenta* obtained in the different culture systems. (**a**) Greenhouse acclimatization after 30 days of cultivation and (**b**) commercial plantation after 90 days of cultivation.

At the acclimatization stage, no differences were observed in the survival rate in the different culture systems evaluated (Table 1). Taro seedlings showed a survival rate of 99% after four weeks of acclimatization (Figure 5a) and were finally transplanted to the field after eight weeks of greenhouse culture (Figure 5b).

## 3. Discussion

### 3.1. Effect of Culture Systems on In Vitro Multiplication

This study demonstrates the efficiency of SABs during in vitro multiplication of taro compared to the semi-solid culture system. The increase in the multiplication rate of *C. esculenta* in SABs could be explained by the assimilation of sugars, nutrients, and growth regulators in the liquid culture medium when remaining in contact with the whole surface of the explant, whereas the low multiplication rate in the semi-solid medium could be due to limited availability of organic and inorganic compounds in the culture medium since it is only in contact with the base of the explant. In addition, in the semi-solid culture systems, there is no loss of apical dominance as occurs in the evaluated bioreactors. In this regard, Nasri et al. [21] note that the loss of apical dominance caused by auxin transport from the apical meristem to the explant base has an effect on the production of new shoots.

The efficiency of the SETIS™ in comparison with other in vitro culture systems has been documented in stevia (*Stevia rebaudiana*) [22], where it doubled the number of shoots compared to the semi-solid culture system. In banana (*Musa* AAA), Bello-Bello et al. [11] achieved a three-fold increase in the number of shoots in the SETIS^TM^ bioreactor compared to the semi-solid system. In sugar cane (*Saccharum* spp.), da Silva et al. [13] obtained five times more shoots in the SETIS^TM^ system compared to the semi-solid medium. The use of TI for cocoyam (*Xanthosoma sagittifolium*) in vitro propagation has been reported by Niemenak et al. [23], demonstrating that the TIB is more efficient for multiplication obtaining 68 shoots during four subcultures. Recently, Arano-Avalos et al. [5] in taro (*C. esculenta*) reported three times the multiplication rate in TIB compared to the semi-solid culture medium.

The low number of roots in the semi-solid medium and ebb-and-flow bioreactor could be due to the fact that the explants are on gel medium in the semi-solid medium and on a polyurethane sponge in the ebb-and-flow bioreactor; these supports probably limit the regeneration of new roots. The increase in root length in the semi-solid medium could be due to the fact that *C. esculenta* is a hydrophytic plant; this condition is affected by the low aeration of the matrix that forms the semi-solid medium and a slow availability of water and nutrients, causing the elongation of the root system in search of water and O_2_.

### 3.2. Chlorophyll Content in Different Culture Systems

Our results showed a higher photosynthetic pigment content in the SETIS^TM^ bioreactor compared to the semi-solid system, the ebb-and-flow bioreactor, and the TIB. In contrast, Arano-Avalos et al. [5] in taro found an increase in photosynthetic pigment content in the TIB compared to the semi-solid medium. Martínez-Estrada et al. [6] reported an increase in chlorophyll accumulation using the ebb-and-flow bioreactor during the in vitro multiplication of *A. andreanum*. Jova et al. [24] in yam (*Dioscorea alata* L. ‘Pacala Duclos’) observed an increase in photosynthetic pigment content using the TIB compared to the constant immersion system and the static liquid system. Aragón et al. [25] in banana obtained higher chlorophyll values in the TIB compared to the semi-solid culture medium.

The increase in chlorophyll contents in *C. esculenta* using the SETIS^TM^ bioreactor is due to its horizontal design made of polycarbonate and polypropylene screw caps positioned in front of the bioreactor. These features facilitate the passage of light into the explants. Irradiance is an important factor with a role in physiological processes such as the synthesis of chlorophyll. The low chlorophyll contents in the semi-solid medium, ebb-and-flow bioreactor and TIB culture systems could be due to the limited light available to the explants because these culture systems were assembled with vertically designed glass vessels and a polypropylene cap on the surface of the culture systems. The effect of light could explain the variation in shoot chlorophyll content within the different culture systems evaluated.

### 3.3. Stomatal Content

The stomatal index (SI) is a physiological parameter that measures the stomatal surface density and is affected by the availability of water (relative humidity (RH), water stress) and light (irradiance, photoperiod, and intensity). This study shows an increase in SI in semi-solid medium and a reduction in TI. This effect could be due to the semi-solid culture system maintains high RH in culture vessels, causing high transpiration rates in plants [10]. The low stomatal index in TI could be due to lower transpiration and temporary water availability every 4 h of immersion frequency. According to Aragón et al. [10], in the TIB, there is constant aeration of the headspace, and the RH is lower. A high SI and percentage of open stomata in the semi-solid medium could be associated with culture hermetic conditions without gas exchange, while a low SI presents an adequate distribution of functional stomata. This fact is important for plant preparation to previous ex vitro conditions.

Stomata are responsible for transpiration through gas exchange between the atmosphere and the plant, and they regulate the water potential in the tissues, thereby maintaining a role in plant homeostasis [26,27]. The stomatal function can be estimated by the percentage of stomata opening and closing. The environmental factors that determine stomatal function are: CO_2_ concentration, RH, water potential, and temperature [28]. In the semi-solid culture system, 4.51% of the stomata were closed, indicating reduced stomatal function. This may have occurred because, being a hermetic system, it has little or no gas exchange (CO_2_ and O_2_), high RH, and high osmotic potential in the culture medium. On the other hand, the SABs increased the percentage of stomatal closure (31–35%), which could be explained by the gas exchange, low RH, and high-water potential in the headspace of the bioreactors. Bello-Bello et al. [11] in banana (*Musa* AAA) report an increase in closed stomata in different TISs (20 to 79%). Martínez-Estrada et al. [6] in anthurium (*Anthurium andreanum* Lind.) report that shoots grown in the liquid medium had the highest percentages of closed stomata (23–29%). According to Asayesh et al. [29] and Bello-Bello et al. [11], SABs with an air supply allow the entry of gases that favor stomatal functioning and chlorophyll synthesis, promoting photo-mixotrophism of plants in vitro. In this regard, Hazarika et al. [30] notes that the increase in the percentage of closed stomata is a physiological indicator of correct stomatal function. The stomata open and close to regulate the amount of water in plant tissue during transpiration and to control gas exchange during photosynthesis [31,32]. According to Regueira et al. [33], ventilation in culture vessels has an effect on the anatomy and physiological processes of plants under in vitro conditions. In vitro propagation in SABs with airflow allows gas exchange to improve photosynthetic capacity in plants prior to the acclimatization stage [5].

### 3.4. Acclimatization

The high survival rates (99%) obtained during acclimatization guarantee the efficiency of the taro in vitro propagation protocol. This result could be due to proper seedling management practices in the greenhouse, such as fertilization, irrigation, and adequate RH and irradiance. These factors contributed to a minimum mortality rate. Similar survival results were obtained by Arano-Avalos et al. [5] when propagating taro in vitro, attributing their findings to the species having minimal requirements during acclimatization. The environment in bioreactor systems provides stress conditions in seedlings for the acclimatization stage [34]. Ahmadian et al. [35] in carnation (*Dianthus caryophyllus* L.) achieved 90% survival using the TIB. Monja-Mio et al. [36] in agave (*Agave tequilana* Weber var. Azul) increased survival up to 96% in a greenhouse when using temporary immersion. A high survival percentage in plants obtained by in vitro propagation is related to adequate stomatal functionality during photorespiration and sufficient photosynthetic pigment content prior to acclimatization [29,37]. The different SABs evaluated favored a low SI and a high % CS compared to the semi-solid medium. This fact suggests that stomatal functionality promotes higher transpiration in plants under temporary immersion conditions.

Different bioreactor models are now available for commercial micropropagation; however, the design is a factor that influences the physiology of plants grown in vitro [11,12]. It is important to carry out studies with different bioreactors due to their increasing availability and variations in cost, bioreactor size, and regeneration pathway used [18,19,38]. Finally, the in vitro plants obtained in this work are being used for the nursery establishment of mother plants. The corms obtained will be used for a commercial plantation for exportation purposes.

## 4. Materials and Methods

### 4.1. In Vitro Establishment

Taro (*Colocasia esculenta* L. Schott) corms were obtained from a commercial plantation located at 19°11′50.9″ N 96°20′16.3″ W. The corms were brought to a greenhouse with natural light of 942 μmol m^−2^ s^−1^, 28 ± 2 °C, and 65 ± 5% relative humidity (RH). After 40 days in the greenhouse, 15 cm apices were extracted and washed with water and Axion^®^ commercial soap (Mission Hills, S.A. de C.V., San José de Iturbide, Guanajuato, Mexico). The apices were taken to the laboratory and immersed in a solution with 2 g L^−1^ of bactericide (Agri-mycin, Pfizer, New York, NY, USA) for 15 min and then rinsed under running water. In a laminar flow hood, the apices were reduced to 5 cm in length and immersed in a 0.54% (*w*/*v*) NaClO solution (6% a.i. Clorox^®^, Monterrey, Mexico) containing two drops of Tween-20^®^ (Sigma-Aldrich^®^ Chemical Company, St. Louis, MO, USA) per 100 mL of solution for 25 min and then immersed in 80% ethanol for 60 s. The apical meristems were extracted and cultured in Murashige and Skoog (MS) [39] establishment medium supplemented with 1 mg L^−1^ of 6-Benzylaminopurine (BAP) (Sigma-Aldrich^®^, St. Louis, MO, USA). The culture medium was adjusted to pH 5.8, and 0.22% (*w*/*v*) Phytagel^®^ (Sigma-Aldrich^®^, St. Louis, MO, USA) was added as a gelling. The culture medium was autoclaved at 121 °C and 116 kPa for 25 min. The explants were incubated at 24 ± 1 °C and a 16-h light photoperiod using LED lamps with an irradiance of 50 μmol m^−2^ s^−1^.

### 4.2. In Vitro Multiplication

Shoots of 2 cm in length from the third subculture (30 d each subculture) were used as explants. The efficiency of the TIB [14], the SETIS™ bioreactor [17], the ebb-and-flow bioreactor [15], and the semi-solid medium culture method as control was compared. All culture systems maintained equal conditions; 50 mL of medium were used per explant in the in vitro multiplication stage, which consisted of MS medium with 3 mg L^−1^ BAP. For the semi-solid system, TIB, and ebb-and-flow bioreactor, 1900 mL glass vessels containing 10 explants were used, maintaining 190 mL of headspace volume per explant. In SETIS™ bioreactors, 29 explants were used to maintain the ratio of 50 mL of culture medium and 190 mL of headspace volume per explant. In SABs, the immersion frequency was 2 min every 4 h, as proposed by Arano-Avalos et al. [5]. In all culture systems, three vessels for semi-solid medium and three bioreactors per SABs were evaluated. The culture vessels and bioreactors were autoclaved at 121 °C and 116 kPa for 30 min. The explants were cultured under the incubation conditions mentioned above. Thirty explants in each culture system were random. After 30 days of culture, the number and length of shoots, number of leaves per shoot, and number and length of roots. In addition, chlorophyll determination, stomatal index, percentage of closed stomata, and survival percentage during acclimatization were determined for each culture system.

### 4.3. Determination of Chlorophyll

Chlorophyll a (chl a), chlorophyll b (chl b) and total chlorophyll (chl t) were determined according to Harborne et al. [40]. Absorbance readings were made using a spectrophotometer (Genesys 10S, Thermo Scientific, Waltham, MA, USA) at 663 and 645 nm for chl a and chl b, respectively. Chlorophyll contents were calculated using the formulas:

Chl a = [[(12.7 × A_663_) − (2.59 × A_645_)] (V)]/(1000 × W)

Chl b = [[((22.9 × A_663_) − (4.70 × A_645_)] (V)]/(1000 × W)

Chl t = chl a + chl b.

where:

A = absorbance.

V = graduation volume in mL^−1^.

W = sample weight in g.

1000 = conversion factor.

### 4.4. Stomatal Index and Percentage of Closed Stomata

Stomatal index (SI) and percentage of closed stomata (% CS) were evaluated in samples of the third leaf of the caulinar apex in the different culture systems. The SI was estimated using the method described by Xu et al. [41]. The underside of developed leaves was used. The samples were observed under a microscope (M5LCD Velab, Co., Pharr, TX, USA). The SI was calculated using the formula:

SI = [NS/(EC + NS)] × 100

where:

SI = stomatal index

NS = number of stomata

EC = number of epidermal cells.

The % CS was calculated using the following formula:

% CS = (CS × 100)/NS

where:

CS: closed stomata

NS: number of stomata

### 4.5. Acclimatization

To assess the effect of survival percentage on different culture systems during acclimatization, 100 seedlings with 3 cm length were evaluated for each culture system. These were grown in a substrate of peat moss, compost, and agrolite (1:1:1 *v*/*v*) using 72-cavity polypropylene trays under a greenhouse with 50% shade mesh at 28 ± 5 °C, 75 ± 10% RH and light of 150 μmol m^−2^ s^−1^. After 40 d in the greenhouse, the survival percentage was determined for each culture system. Finally, the seedlings were transplanted into 38-cavity polypropylene trays and kept in an unshaded greenhouse at 28 ± 2 °C, 60 ± 10% RH and light of 950 μmol m^−2^ s^−1^.

### 4.6. Experimental Design and Statistical Analysis

All experiments were distributed in a fully random design and were replicated three times. Data were subjected to an ANOVA and comparison of means according to Tukey (*p* ≤ 0.05) using SPSS^®^ software (version 22). Percentage data were transformed with the formula Y = arcsine (√(x/100)), where x is the percentage value.

## 5. Conclusions

In conclusion, the results obtained show that the different culture systems evaluated had an effect on the in vitro multiplication of taro, with no effect on the survival rate during acclimatization. The SABs increased the shoot regeneration rate compared to the semi-solid medium, with the SETIS™ bioreactor showing the highest multiplication rate and the highest chlorophyll content; however, other bioreactor alternatives are also available that allow for semi-automation and cost reduction for micropropagation of *C. esculenta*. Plants obtained in this study were invigorated and high-quality phytosanitary. Similar studies should be performed in other cultivars of *C. esculenta*.

## Figures and Tables

**Table 1:** Effect of culture systems on the in vitro multiplication and ex vitro survival of taro (*Colocasia esculenta*) after 30 days of culture.

Culture Systems	Shoots/Explant	Shoot Length (cm)	Leaves/Shoot	Roots/Explant	Root Length (cm)	Survival (%)
Semi-solid medium	6.10 ± 0.31 ^c^	3.80 ± 0.22 ^b^	2.70 ± 0.21 ^c^	5.80 ± 0.53 ^c^	5.60 ± 0.33 ^a^	99.66 ± 33 ^a^
TIB	21.70 ± 1.14 ^b^	5.50 ± 0.33 ^a^	3.70 ± 0.30 ^a^	21.30 ± 1.28 ^b^	3.00 ± 0.33 ^b^	99.33 ± 33 ^a^
SETIS	36.00 ± 1.26 ^a^	4.50 ± 0.30 ^a^	3.60 ± 0.22 ^a^	41.60 ± 2.50 ^a^	2.80 ± 0.27 ^b^	99.33 ± 33 ^a^
Ebb-and-flow bioreactor	20.00 ± 0.79 ^b^	2.50 ± 0.22 ^c^	3.10 ± 0.23 ^b^	2.50 ± 0.22 ^c^	1.40 ± 0.14 ^c^	99.00 ± 57 ^a^

Means (±standard error) with different letters are significantly different (Tukey, *p* ≤ 0.05). TIB: temporary immersion bioreactor; SETIS: SETIS™ bioreactor.

## Data Availability

Not applicable.
